# β-Galactosidase-Activatable Nile Blue-Based NIR Senoprobe for the Real-Time Detection of Cellular Senescence

**DOI:** 10.1021/acs.analchem.2c04766

**Published:** 2022-12-29

**Authors:** Beatriz Lozano-Torres, Alba García-Fernández, Marcia Domínguez, Félix Sancenón, Juan F. Blandez, Ramón Martínez-Máñez

**Affiliations:** † Instituto Interuniversitario de Investigación de Reconocimiento Molecular y Desarrollo Tecnológico (IDM), Universitat Politècnica de València-Universitat de València, Camí de Vera S/N, Valencia 46022, Spain; ‡ Unidad Mixta UPV-CIPF de Investigación en Mecanismos de Enfermedades y Nanomedicina, Universitat Politècnica de València, Centro de Investigación Príncipe Felipe, C/ Eduardo Primo Yúfera 3, Valencia 46012, Spain; § CIBER de Bioingeniería, Biomateriales y Nanomedicina, Av. Monforte de Lemos, 3-5, Pabellón 11, Planta 0, Madrid 28029, Spain; ∥ Unidad Mixta de Investigación en Nanomedicina y Sensores, Universitat Politècnica de València, IIS La Fe, Av. Fernando Abril Martorell, 10, Torre A 7a̲ planta, Valencia 46026, Spain

## Abstract

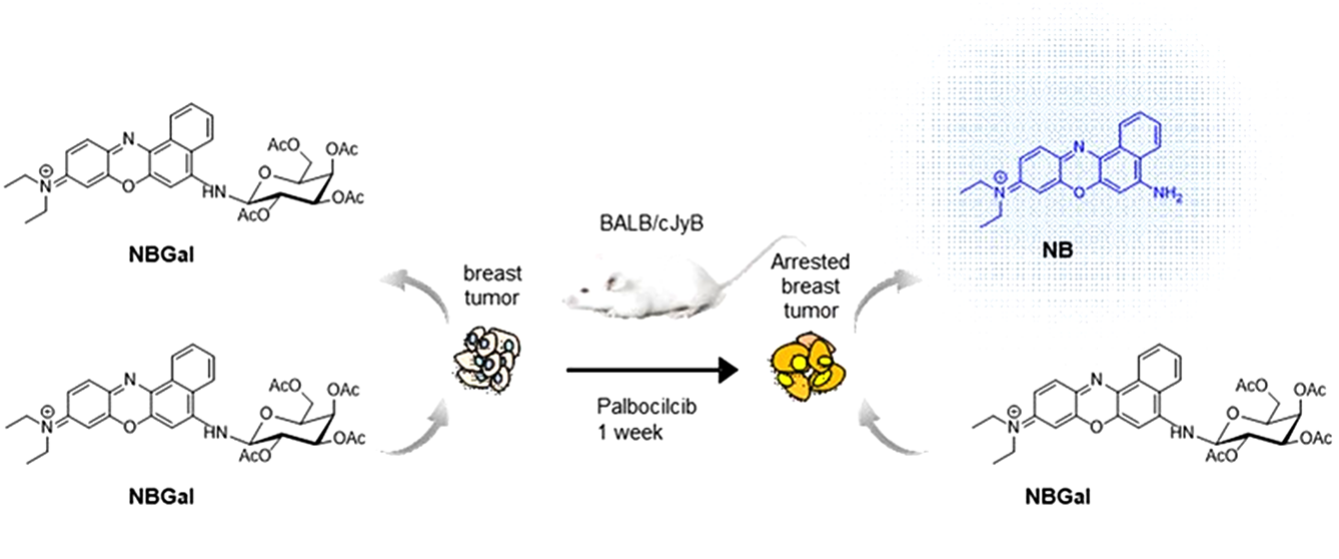

Cellular senescence is a stable cell cycle arrest in response to stress or other damage stimuli to maintain tissue homeostasis. However, the accumulation of senescent cells can lead to the progression of various senescence-related disorders. In this paper, we describe the development of a β-galactosidase-activatable near-infrared (NIR) senoprobe, **NBGal**, for the detection of senescent cells based on the use of the FDA-approved Nile blue (**NB**) fluorophore. **NBGal** was validated in chemotherapeutic-induced senescence cancer models *in vitro* using SK-Mel 103 and 4T1 cell lines. *In vivo* monitoring of cellular senescence was evaluated in orthotopic triple-negative breast cancer-bearing mice treated with palbociclib to induce senescence. In all cases, **NBGal** exhibited a selective tracking of senescent cells mainly ascribed to the overexpressed β-galactosidase enzyme responsible for hydrolyzing the **NBGal** probe generating the highly emissive **NB** fluorophore. In this way, **NBGal** has proven to be a qualitative, rapid, and minimally invasive probe that allows the direct detection of senescent cells *in vivo*.

Senoprobes, molecules specifically designed to detect senescent cells, have gained interest due to their potential use for monitoring cellular senescence associated with multiple diseases. Senescence is a cellular response to stress and damage stimuli to maintain tissue homeostasis.^1,2^ However, the persistent presence of senescent cells can promote chronic inflammation, tissue dysfunctionality, and tumorigenesis, thus contributing to different diseases such as fibrosis, tissue aging, tumorigenesis, and metastasis.^3,4^

Cells can undergo senescence in response to different stimuli, such as replicative exhaustion, the inhibition of tumor suppressor genes, the activation of oncogenes, the accumulation of DNA damage, and the presence of reactive oxygen species (ROS), or after exposure to certain drugs like chemotherapeutics, among others.^5,6^ Senescent cells are characterized by several cellular mechanisms and alterations.^7^ Once the senescence mechanism has been triggered, the arrest in the cell cycle becomes irreversible, mainly regulated by two pathways: (i) the interaction between the proteins p53/p21, inhibitors of cyclin-dependent kinases (CDK) and (ii) the interaction between the p16Ink4a/retinoblastoma (pRb) proteins, which are related to the expression of genes, necessary for cell cycle progression.^8^ Among these changes, senescent cells exhibit high levels of phosphorylated retinoblastoma proteins, p53, p16, and p21.^9,10^ Besides, as a result of their uncontrolled secretory activity, cytokines, chemokines, and metalloproteinases generated constitute the characteristic senescence-associated secretory phenotype of senescent cells (SASP).^11^ Changes in the morphology with increased size and irregular shape are also characteristic of senescent cells, mainly attributed to an increased lysosomal compartment. As a result, senescent cells present a high accumulation of lipofuscin granules and the overexpression of many lysosomal enzymes.^12,13^ Among them, the overexpression of the lysosomal enzyme β-galactosidase has been established as a biomarker for the detection of senescent cells, referred to as senescence-associated β-galactosidase (SA-βGal).^14^ SA-βGal is encoded by the GLB1 gene, but the mechanisms involved in this overproduction are currently unknown.^15^

Due to the harmful role of senescence, the selective elimination of senescent cells, referred to as senolysis, has become an effective strategy in many senescence-related disorders, including cancer and aging-associated diseases.^16−18^ On the other hand, the guided activation of senescence via therapy-induced senescence (TIS) has been employed in clinics to arrest tumor growth. Recently, the effectiveness of the combination of TIS with the subsequent elimination of senescent cells has been studied in preclinical models.^19−22^ These results argue the importance of the clinical need for developing universal probes for the detection of senescent cells in living organisms with the ability to monitor senescence burden.^23,24^ However, the development of suitable techniques able to carry out the *in vivo* monitoring of senescence is still an unresolved problem.

In the last few years, the application of molecular probes in biomedicine has become a topic of high importance, as they are being used in the detection and monitoring of individual molecules or biomarkers. These kinds of probes have also been used to study molecular interactions at cellular and subcellular levels.^25^ Molecular imaging techniques allow *in vivo* imaging that is employed in the characterization of molecules and biological processes to improve clinical diagnosis and the development of drugs in many pathologies.^26^ Among the techniques that allow molecular imaging *in vitro* and *in vivo*, the use of fluorescent probes shows high appeal due to the ability to obtain direct images using a fluorescence imaging instrument. These types of equipment are inexpensive instruments commonly found in scientific facilities.^27^

Based on optical molecular imaging and related to senescence tracking, SA-βGal overexpression has been extensively used for the design of commercial kits (such as X-Gal and Spider-βGal) for cellular senescence detection. However, the use of these commercial kits for *in vivo* tracking is limited.^28,29^ In the literature, some fluorescent probes have been recently described to overcome these limitations.^30−33^ These probes usually consist of a fluorophore (acting as a signaling unit) covalently linked to a galactose molecule (which acts as a recognition unit). The covalent attachment of these two units, through N or O glycosidic bonds, induces a marked decrease in the fluorescence emission of the fluorophore that is recovered upon the hydrolysis of the N or O glycosidic bonds promoted by the SA-βGal enzyme. Despite most of these senoprobes having been successfully applied in the detection of senescent cells, some of them still present some drawbacks. For instance, the majority of probes developed for the detection of senescence based on SA-β-Gal overexpression are only useful for detection *in vitro* and *ex vivo* after the animals are sacrificed, and there are relatively few probes able to monitor senescence *in vivo* using fluorescence imaging instrumentation.^34−38^ The quantitative precision and sensitivity of probes are also limited due to autofluorescence from biological tissues in the lower visible fluorescence ranges.^24,39,40^ To avoid autofluorescence problems working with biological samples, it is convenient to use fluorophores with emission wavelength and excitation in the near-infrared (NIR) domain.^25,41^ It is well known that the use of radiation in the NIR range allows greater penetration into the sample, thereby managing to evaluate information on less superficial structures. The use of NIR fluorophores also involves a reduction in cell damage since lower energy radiations are used to excite the probes.^42^ Among NIR fluorophores available, Nile blue (**NB**) exhibits remarkable features as an *in vivo* imaging agent. **NB** is an aromatic planar fluorophore with a phenoxazine structure that is commercially available and easy to be modified chemically. On the other hand, **NB** has been approved by the Food and Drug Administration (FDA) for human use for some indications.^25,43,44^

Based on the above, and taking into account our interest in the synthesis of sensors for senescent cell detection,^31,45^ here we report the development of a β-galactosidase-activatable NIR senoprobe (**NBGal**) for monitoring of cellular senescence (Scheme 1). The probe is based on the use of the **NB** fluorophore linked, through a galactosamine bond, to a galactose derivative (Figure 1a). While **NBGal** is poorly emissive, the hydrolysis of the galactosamine bond in the presence of the overexpressed SA-βGal in senescent cells results in the release of the **NB** dye with the subsequent enhancement in the fluorescence signal. The **NBGal** probe is validated in chemotherapy-induced cancer models *in vitro*, *in vivo*, and *ex vivo*, confirming its potential for monitoring cellular senescence in living organisms.

**Figure 1:**
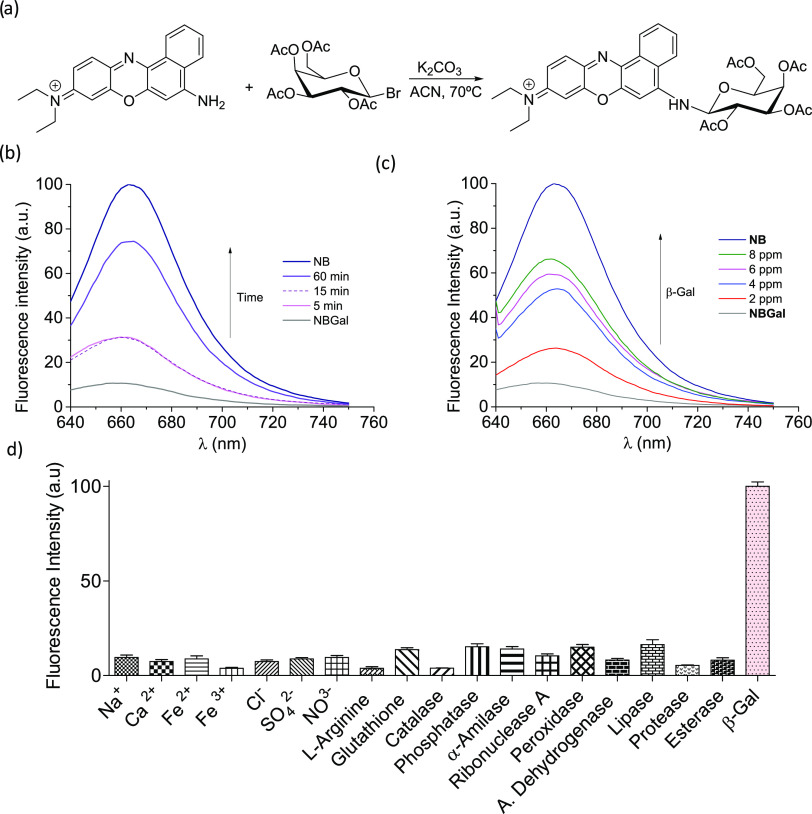
(a) Synthesis of an **NBGal** probe. (b) Fluorescence emission of **NBGal** in the presence of the β-Gal enzyme (2 ppm) as a function of time and (c) as a function of enzyme amount after 10 min of incubation in phosphate-buffered saline (PBS) (pH 7)—dimethylsulfoxide (DMSO) (0.01%). (d) Fluorescence emission of **NBGal** (10^–6^ M) in PBS (pH 7)—DMSO (0.01%) in the presence of different cations, anions, neutral molecules, and enzymes. Values are expressed as mean ± standard deviation (SD) (*n* = 3).

**Scheme 1:**
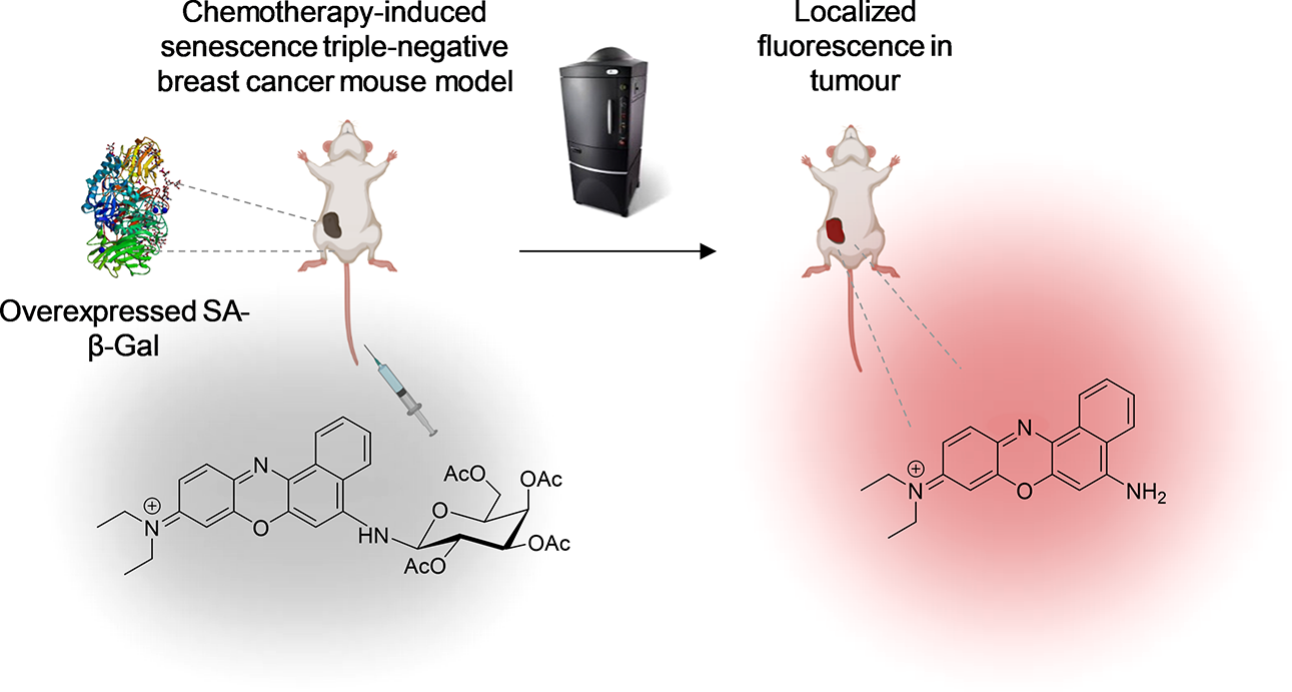
Representation of the β-Gal-Activatable **NBGal** Probe for the *In Vivo* Monitoring of Cellular Senescence

## Results and Discussion

### Synthesis and Characterization of the **NBGal** Probe

**NBGal** was synthesized through a nucleophilic substitution reaction between 2,3,4,6-tetra-*O*-acetyl-α-D-galactopyranosyl bromide (Gal) and the **NB** fluorophore in the basic medium (Figure 1a). Final product **NBGal** was characterized by ^1^H NMR, ^13^C NMR, and high-resolution mass spectrometry (HR-MS) (Figures S1–S3). The UV–visible spectra of **NBGal** and **NB** in PBS (pH 7)—DMSO (0.01%) solution showed the absorbance bands centered at 565 and 636 nm, respectively. After excitation of the **NB** solution in PBS (pH 7)—DMSO (0.01%), a marked emission band at 666 nm (Φ_**NB**_ = 0.004) was found, whereas the **NBGal** probe showed a weak emission band centered at 660 nm (Φ_**NBGal**_ = 0.0015) (Figure S4).

Besides, taking into account the fact that **NB** is an acid–base indicator, the emission of the fluorophore and probe in H_2_O–DMSO (0.01%) solutions at different pH values was studied (Figure S5). The obtained results indicated that **NB** emission decreased at neutral and slightly basic pH (ca. 70% at pH 9.0). However, the **NB** fluorophore shows a strong fluorescence signal at a slightly acidic pH (5–6), characteristic of the lysosomal compartment (Figure S5). Besides, due to the planar character of **NB**, a marked emission quenching was observed for PBS (pH 7)—DMSO (0.01%) solutions of concentrations higher than 1.0 × 10^–4^ M (Figure S6), which was ascribed to π-stacking interactions between the planar **NB** molecules. In sharp contrast, the emission of **NBGal** was not quenched at these concentrations (Figure S6), probably due to ineffective π-stacking interactions because of the presence of galactose in the probe structure.

To test the sensing ability of **NBGal**, the emission of the probe was studied in the presence of human β-Gal. Results showed that the weak emission at the band at 660 nm of **NBGal** in PBS (pH 7)—DMSO (0.01%) was markedly enhanced with time in the presence of a human β-Gal enzyme (6.8-fold after 60 min, Figure 1b). Besides, the increase of fluorescence intensity at 660 nm was β-Gal-concentration-dependent (Figure 1c). The emission enhancement observed was ascribed to the β-Gal-induced hydrolysis of the galactosamine bond presented in **NBGal** and the consequent formation of **NB**. Changes in the emission of PBS (pH 7)—DMSO (0.01%) solutions of **NBGal** in the presence of selected potential interferents were studied. For this purpose, solutions of Na^+^, Ca^2+^, Fe^2+^, Fe^3+^, Cl^–^, SO_4_^2–^, NO_3_^–^, l-arginine, reduced glutathione, catalase, phosphatase, α-amylase, ribonuclease A, peroxidase, alcohol dehydrogenase, lipase, protease, esterase, and human β-Gal at a final concentration of 150 μM for cations, anions, and small peptides, or 150 μg/μL for enzymes were added to solutions of **NBGal**, and the emission intensity at 660 nm was measured after 60 min. As shown in Figure 1d, only the human β-Gal enzyme induced a marked emission enhancement at 660 nm. This emission enhancement is ascribed to the β-Gal-induced hydrolysis of the galactosamine bond in **NBGal** (leaving the acetyl moieties unhydrolyzed) with the subsequent formation of a highly emissive **NB** fluorophore. However, the esterase enzyme hydrolyzes acetyl moieties in **NBGal** but is unable to hydrolyze the galactosamine bond and, as a result, **NB** is not produced, and low emission is observed. Finally, a limit of detection for **β**-Gal of 6.86 ng/mL (2.33 U/mL) was measured by monitoring the fluorescence emission of the **NBGal** probe at 666 nm after 60 min of enzyme addition (Figure S7).

### *In Vitro* Validation of the **NBGal** Probe

**Figure 2:**
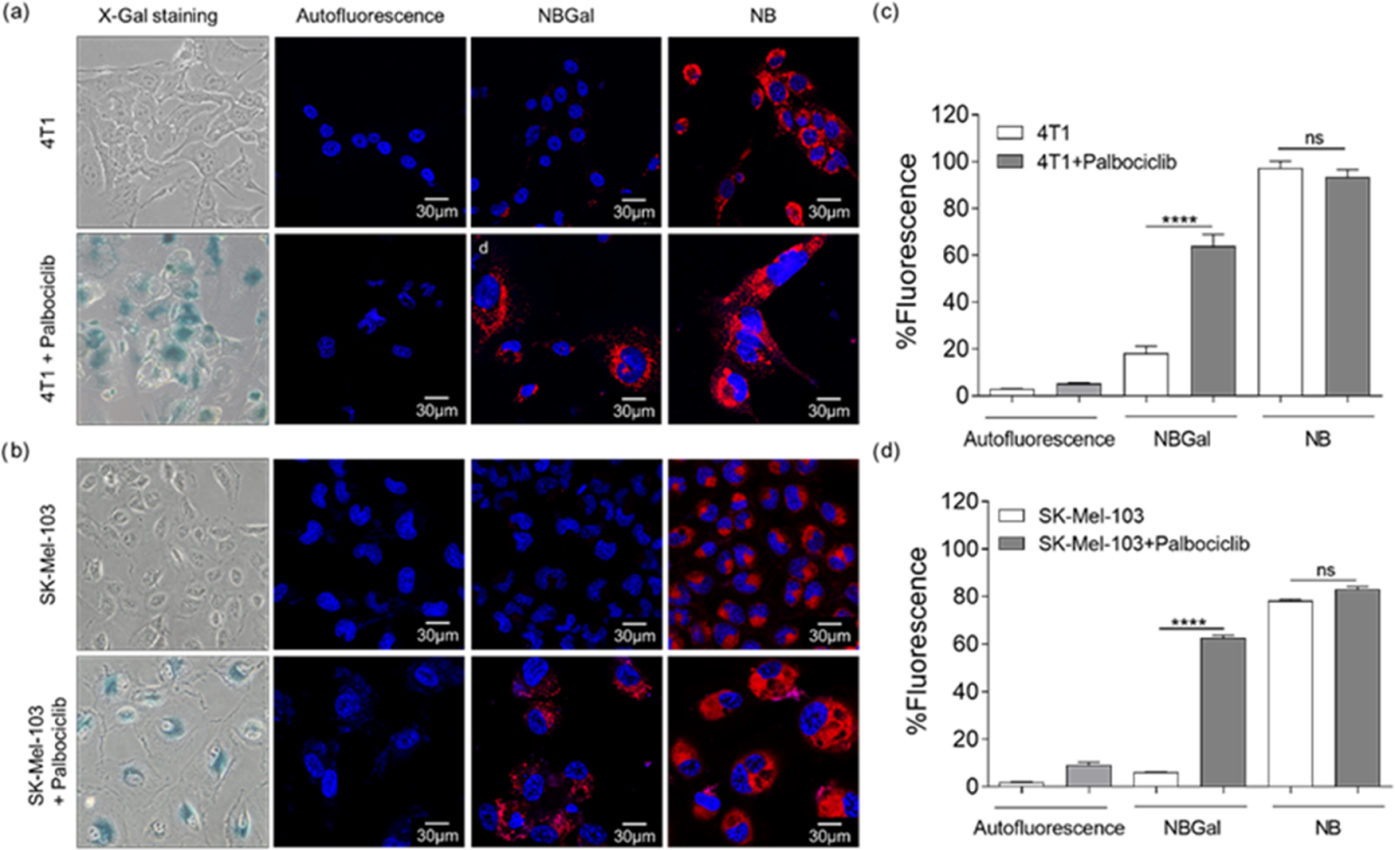
(a) Images of control 4T1 (up) and senescent 4T1 cells (4T1 cells treated with palbociclib) (down). (b) Images of control SK-Mel-103 (up) and senescent SK-Mel-103 cells (SK-Mel-103 cells treated with palbociclib) (down). From left to right: X-Gal staining images and confocal images from nontreated cells, incubated with **NBGal** (1.25 μM) and **NB** (1.25 μM), respectively. (c) Quantification of the fluorescence emission intensity relative to the cell surface of control and senescent palbociclib-treated 4T1 cells and (d) of control and senescent palbociclib-treated SK-Mel-103 cells, respectively. The results exhibited representative data from three independent studies (*n* = 3), and values are expressed as mean ± SD. Statistical analysis was assessed by applying two-way analysis of variance (ANOVA) with multiple comparisons (****p < 0.001).

The specificity of **NBGal** to detect the presence of senescent cells *in vitro* was demonstrated in chemotherapy-induced senescence cancer models. For this purpose, breast cancer 4T1 cells and human melanoma SK-Mel-103 cells were incubated with palbociclib (5 μM), a CDK4/6 inhibitor responsible for cell cycle arrest, for one week. Palbociclib-treated cells showed the typical blue signal upon X-Gal treatment in comparison with proliferative cells, which corroborated the induction of senescence in both cell lines (Figure 2a,b). Additionally, we also confirmed that neither control nor senescent cells incubated in a wide range of concentrations with **NBGal** exhibited any sign of significant toxicity in the working dose (Figure S8).

Finally, confocal assays were carried out to assess the selective activation of the **NBGal** probe in senescent cells. A strong fluorescence signal for senescent 4T1 (Figure 2a) and senescent SK-Mel-103 (Figure 2b) cells treated with **NBGal** was observed when compared to proliferating cells. In contrast, cells incubated with **NB** showed a high fluorescence signal in all cases, which corroborated that the **NB** fluorophore does not exhibit selectivity for senescence cell detection. Fluorescence quantification of confocal images confirmed the enhanced fluorescence emission of **NBGal** in palbociclib-treated 4T1 cells compared to control 4T1 cells (ca. 4-fold) (Figure 2c) as well as in senescent SK-Mel-103 cells compared to control SK-Mel-103 cells (ca. 10-fold) (Figure 2d). Besides, autofluorescence was discarded for 4T1 or SK-Mel-103 control and senescent cells. Moreover, confocal images at different magnifications (20×) were also obtained (Figure S9). Overall, the results confirm the *in vitro* detection of senescent cells by **NBGal**.

### *In Vivo* Validation of the **NBGal** Probe

**Figure 3:**
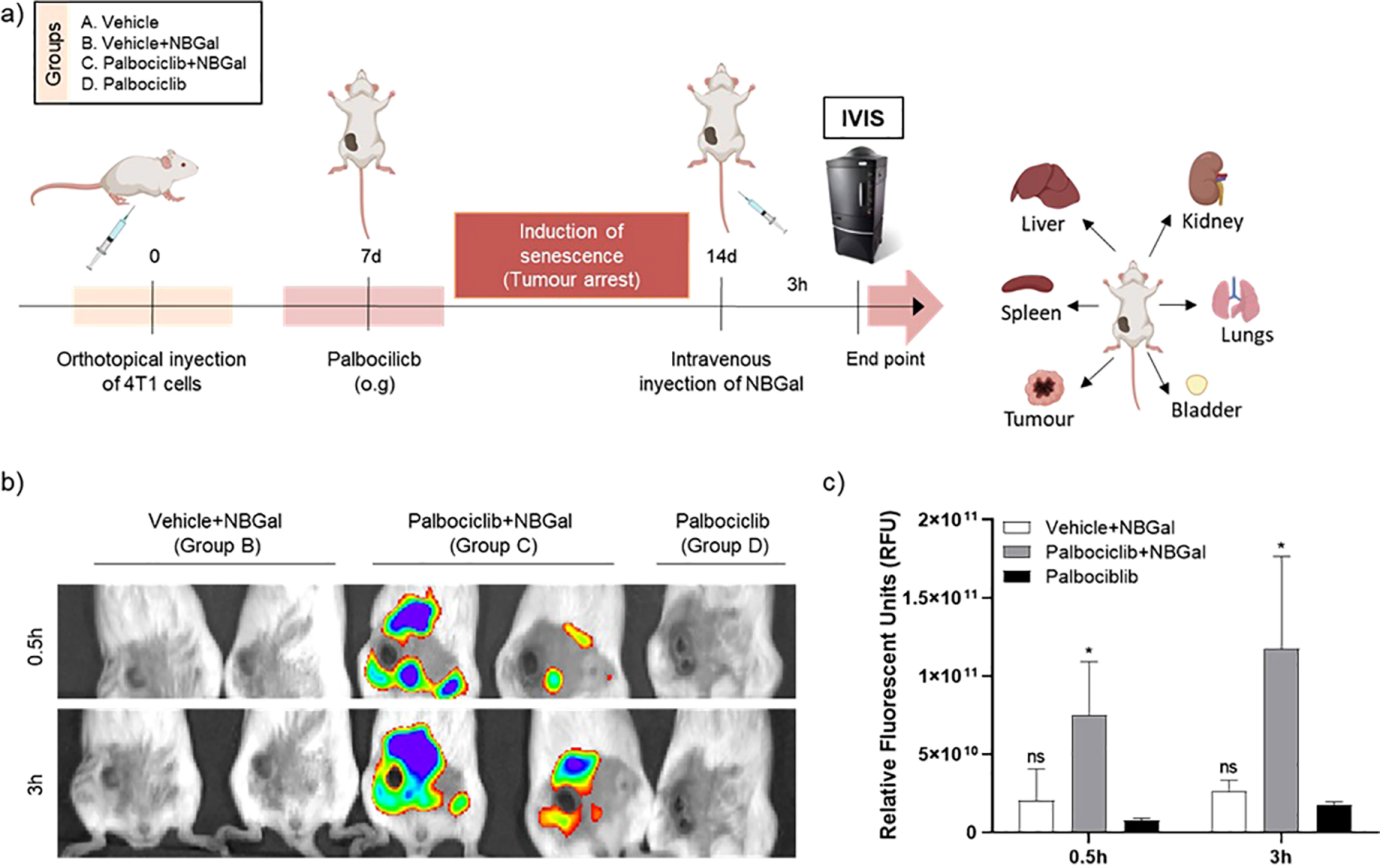
(a) Palbociclib-induced senescent cancer model. For tumor generation, 4T1 cells were injected subcutaneously into the left mammary fat pad of female BALB/cByJ mice. Mice were subsequently treated with palbociclib (100 mg/kg) or vehicle by daily oral gavage (og) for 7 days. Mice were then divided into four groups of 5 animals: (A) Vehicle; (B) Vehicle + **NBGal**; (C) palbociclib + **NBGal**; and (D) palbociclib. **NBGal** was intravenously injected into the tail vein, and mice were monitored by IVIS imaging. Then, mice were sacrificed, and organs and tumors were collected. (b) Representative IVIS images of 4T1 tumor-bearing mice at 0.5 and 3 h post-injection. From left to right: (B) Vehicle + **NBGal**; (C) palbociclib + **NBGal**; and (D) palbociclib. (c) Quantification of average radiance intensity from IVIS images in the tumor zone shown in (b). The results are expressed as mean ± SD, and statistical analysis was performed by applying Two-way ANOVA with multiple comparisons (**p < 0.01 and ****p < 0.001).

Once the preferential hydrolysis of **NBGal** in senescent cells was assessed *in vitro*, the probe was validated *in vivo* in a chemotherapy-induced senescence breast cancer mouse model (Figure 3a). In addition to the potential of monitoring senescence in cancer therapy, this model provides a standard of controlled senescence burden *in vivo* as SA-β-Gal activity is mainly found in tumors upon treatment with palbociclib. For tumor development, BALB/cByJ female mice were orthotopically injected with the 4T1 breast cancer cells (0.5 × 10^6^ cells).

Then, mice were anesthetized and monitored in real-time using an *in vivo* imaging system (IVIS) at 0.5 and 3 h post **NBGal** treatment. IVIS images revealed that mice from groups A (data not shown), B, and D showed negligible fluorescence in the tumor zone, while a strong fluorescence signal was observed for group C, administered both with palbociclib and **NBGal** (Figure 3b). The quantification of the relative values of radiance (p/s/cm^2^/sr × 10^11^) from IVIS images confirmed this effect. An enhancement of fluorescence, ca. 4-fold at 0.5 h and near to 10-fold at 3 h, was observed for palbociclib + **NBGal-**treated mice (Group C) when compared to mice treated with vehicle + **NBGal** (group B) (Figure 3c). Remarkably, the fluorescence signal only increased over time in animals treated with palbociclib and **NBGal**, whereas in animals treated with the vehicle and **NBGal**, the probe remained inactive, and nearly zero emission was observed during the experiment. Therefore, these findings confirmed that upon *in vivo* administration, the nonemissive **NBGal** enters the bloodstream while remaining inactive unless taken up by senescent cells.

**Figure 4:**
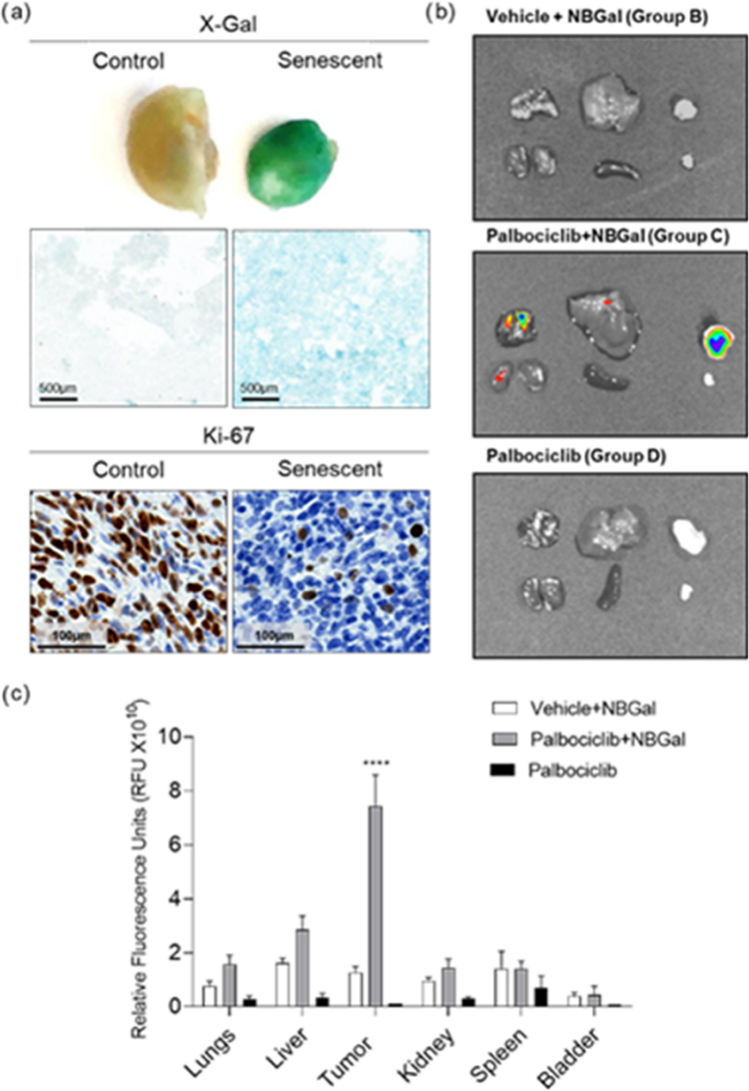
(a) Representative images of tumors from BALB/cByJ stained for SA-β-Gal activity and the proliferative markers Ki67 in mice treated with vehicle (left) or palbociclib (right). (b) Representative IVIS images of organs and tumors from mice treated with vehicle + **NBGal** (group B), palbociclib + **NBGal** (group C), and palbociclib (group D). From left to right and up to bottom: lungs, liver, tumor, kidneys, spleen, and bladder. (c) Quantification of IVIS images shown in (b). The results are expressed as mean ± SD (*n* = 5), and statistical analysis was performed by applying Two-way ANOVA with multiple comparisons (****p < 0.001).

After confirming the optical detection of senescent tumors *in vivo* using **NBGal**, mice were euthanized, and different organs (i.e., lungs, liver, kidney, spleen, and bladder) and tumors were harvested. 4T1 breast tumors treated with palbociclib revealed the specific induction of senescence by X-Gal staining in the whole tumor and tumor sections, showing the typical blue staining (Figure 4a, up).

Moreover, tumors from mice treated with palbociclib exhibited a significant decrease of the Ki67 proliferative maker compared to those from mice treated with the vehicle (Figure 4a, down). IVIS images from organs and tumors were also acquired and quantified. Palbociclib-treated mice (group D) did not present any autofluorescence signal in the different analyzed organs and senescent tumors. Mice injected with the vehicle + **NBGal** probe (group B) did not exhibit any fluorescence signal in tumors and collected organs. In contrast, in palbociclib-treated mice injected with the **NBGal** probe (group C), a strong fluorescence signal was registered in the senescent tumors, and only a basal fluorescence was observed in organs (Figure 4b). The quantification of the IVIS images confirmed these observations (Figure 4c); i.e., a significant fluorescence signal was only detected in the senescent tumors from palbociclib + **NBGal**-treated mice and not in their untreated counterparts or the other organs.

Overall, these results corroborate that **NBGal** renders inactive until its preferential activation in senescent tumor cells, in which the overexpressed β-Gal hydrolyzes the galactosamine bond and release the **NB** dye with the subsequent enhancement in the fluorescence signal. Our studies also demonstrate the selective targeting of senescent cells *in vitro* and *in vivo* by **NBGal**.

## Conclusions

We herein report the development of a new **NBGal** senoprobe based on the conjugation of a galactose derivative with the NIR fluorescent dye **NB**. The ability of **NBGal** for the detection of cellular senescence *in vitro* was assessed in 4T1 (murine breast cancer) and SK-Mel-103 (human melanoma) palbociclib-induced senescent cell lines. The significant fluorescence signal is only observed in senescent cells compared to proliferative cells. Moreover, real-time *in vivo* detection of senescence with the **NBGal** probe was validated in a chemotherapy-induced senescence breast cancer mouse model. Female mice orthotopically injected with 4T1 cells to generate breast tumors and treated with palbociclib were used. *In vivo*, IVIS images exhibited a remarkable fluorescence signal only in tumor-bearing animals treated with palbociclib and **NBGal**, whereas no significant fluorescence signal was found in 4T1 tumor-bearing mice treated with vehicle or **NBGal** alone. Real-time images from mice and *ex vivo* analysis confirmed the presence of a significant fluorescence signal only in senescent tumors. Overall, these results demonstrate the ability of **NBGal** as a senoprobe for the specific monitoring of cellular senescence *in vivo*. Senoprobes like **NBGal** are necessary tools for a number of applications, such as monitoring senescence burden, following studies in therapy-induced senescence cancer models, and following up on the efficacy of senolytic treatments. In this scenario, **NBGal** has proven to be a qualitative, rapid, and minimally invasive analysis method for real-time senescence detection. In addition, **NB** is an organic dye approved by the FDA for human use, which might facilitate further studies for clinical applications. Table S1 lists other recently published **β**-Gal probes capable of detecting senescent cells *in vitro* and *in vivo* models. Taking it all together, there is no doubt that clinical uses of nanoprobes for senescence detection will emerge in the next years for different applications.

## Experimental Section

### Synthesis of **NBGal**

#### (2*R*,3*S*,4*S*,5*R*,6*R*)-2-(Acetoxymethyl)-6-(((*Z*)-9-(diethylamino)-5*H*-benzo[*a*]phenoxazin-5-ylidene)amino)tetrahydro-2*H*-pyran-3,4,5-triyl-triacetate

Commercially available **NB** perchlorate (208 mg, 0.5 mmol), acetobromo-α-d-galactose (616 mg, 1.5 mmol), and K_2_CO_3_ (414 mg, 3 mmol) were charged into a round-bottomed flask. Upon purging with argon atmosphere, anhydrous acetonitrile (20 mL) was added, and the reaction mixture was stirred for 4 h at 70 °C. Afterward, the solvent was removed under vacuum pressure to dryness. The residue was purified by column chromatography using silica gel with ethyl acetate/hexane 1:10 v/v as an eluent. The product was isolated as a purple-red solid (188 mg, 0.29 mmol, 58% yield). The product was characterized by ^1^H NMR, ^13^C NMR, and HR-MS (see Figures S1–S3).

### Hydrolysis of **NBGal**

The hydrolysis reaction of the **NBGal** probe by the human β-Gal enzyme was analyzed by fluorescence spectroscopy. For this purpose, 2 μL (0.8 μg/μL) of the human β-Gal enzyme was added to PBS (pH 7)—DMSO (0.01%) solutions of **NBGal** (1.0 × 10^–6^ M), and the emission spectrum at 666 nm (λ_exc_ = 636) was recorded as a function of time. The emission band of the hydrolysis product was compared with the NB fluorophore solution in the same conditions (Figure 1b). In a similar experiment, the hydrolysis of **NBGal** was monitored after the addition of different enzyme amounts in the range from 0 to 5 μL (0.8 μg/ μL). In this case, a different amount of β-Gal enzyme was added to PBS (pH 7)—DMSO (0.01%) solutions of **NBGal** (1.0 × 10^–6^ M). After 10 min of the addition of the enzyme, samples were recorded at 666 nm upon excitation at 636 nm.

### Interferents

To determine the specificity and selectivity of the probe through β-Gal detection, fluorescence emission at 666 nm of **NBGal** solutions (1 mL, 1.0 × 10^–6^ M) was measured in the presence of cations (150 μM), anions (150 μM), small peptides (150 μM), proteins (150 μg/mL), and enzymes (150 μg/mL) (Figure 2d) at 37 °C after 1 h (λ_ex_ = 636 nm). The references of the interferent used were: Na_2_SO_3_, CaCl_2_, FeS, Fe(NO_3_)_3_, l-arginine, reduced glutathione, catalase from bovine liver, phosphatase from potato, α-Amilase from *Aspergillus oryzae*, ribonuclease A from the ovine pancreas, peroxidase from radish, glucose oxidase from *Aspergillus niger*, alcohol dehydrogenase from *Saccharomyces cerevisiae*, lipase from porcine pancreas, protease from *Streptomyces griseus*, and esterase from the porcine liver, all from Sigma Aldrich, and human β-Galactosidase from Bio-Techne.

### Cell Lines

SK-Mel-103 (human melanoma cancer cells) and 4T1 (murine triple-negative breast cancer cells) were obtained from ATCC. Cells were maintained in a DMEM supplemented with 10% FBS and incubated in 20% O_2_ and 5% CO_2_ at 37 °C. Cells were treated with palbociclib (5 μM) for one week to induce the senescence phenotype.

### X-Gal Staining Assay

The induction of senescence after palbociclib treatment in SK-Mel-103 and 4T1 cells was assessed using a Senescence β-Galactosidase Staining Kit (#9860, Cell Signaling) following the manufacturer′s instructions.

### *In Vitro* Viability Assays

Proliferating and senescent SK-Mel-103 and 4T1 cells were seeded in flat-bottom-clear 96-well plates at 4000 and 6000 cells/well, respectively. After 24 h, cells were treated with serial dilutions of **NBGal**. Viability was assessed 48 h later using a CellTiter-Glo luminescent cell viability assay following the instructions of the kit. Raw data were obtained by measuring luminescence in a VICTOR multilabel plate reader (PerkinElmer). To calculate the % of viability in each assay, we normalize each value to the average of the respective control group (untreated cells) of proliferative or senescent cells and finally multiply by 100. The results are expressed as mean ± SD from tree-independent studies (*n* = 3).

### Confocal Assays

Proliferative and senescent cells were seeded in the 96-well Black OptiPlate at a concentration of 5000 cells/well for SK-Mel-103 and 4T1 cells, respectively. After 24 h, cells were treated with **NBGal** and **NB** at 1.25 μM (DMEM with 0.1% DMSO) for 2 h. Then, cells were washed with PBS, and Hoechst 33348 (2 μg/mL) was added for nuclei staining. Confocal fluorescence images were taken on a Leica TCS SP8 AOBS using 638 nm as excitation wavelength. Images were obtained at 20× magnification, and the fluorescence intensity was analyzed cell by cell from different fields (randomly selected) conformed by 50–100 cells in each one, using ImageJ software. To calculate the % of fluorescence intensity in each assay, we normalize each value (proliferative and senescent) to the average of the higher fluorescence signal and finally multiply by 100. The results are expressed as mean ± SD from three independent studies (*n* = 3). A p-value below 0.05 was considered statistically significant and indicated with asterisks: *p < 0.05, **p < 0.0, ***p < 0.005, and ****p < 0.001. To obtain images at high magnification (63×), proliferative and senescent cells were seeded at a concentration of 250,000 cells/well for SK-Mel-103 and 4T1 cells, respectively. After 24 h, cells were treated with **NBGal** and **NB** at 1.25 μM for 2 h. Finally, cells were washed with PBS, coverslips were mounted, and Hoechst 33348 (2 μg/mL) was added for nuclei staining. Confocal fluorescence images were taken on a Leica TCS SP8 AOBS. The results showed representative images from three independent studies (*n* = 3).

### Animal Models

Balb/cByJ mice were maintained at the Spanish Research Center Principe Felipe (CIPF) under the recommendations of the Federation of European Laboratory Animal Science Associations (FELASA). To generate breast tumors, 4T1 cells were orthotopically injected subcutaneously in the left mammary pad of 28- to 34-week-old BALB/cByJ female mice at a concentration of 0.5 × 10^6^ cells in a volume of 100 μL. Tumor volume was measured every 2 days with a caliper and calculated as *V* = (*a* × *b*^2^)/2, where *a* is the longer and *b* is the shorter of two perpendicular diameters. After one week, the mice were separated into four groups (*n* = 5/group). Mice from groups A and B were treated with vehicle, whereas animals in groups C and D were treated daily by oral gavage (og) with palbociclib to induce senescence. Palbociclib (100 mg/kg) or vehicle was administered by daily oral gavage for 7 days dissolved in 50 mM sodium lactate at pH 5. Then, **NBGal** was intravenously administered at a concentration of 5 mg/mL in DMEM (5% DMSO) in a volume of 200 μL to groups B and C. The fluorescence emission in animals was monitored in real-time after 30 min and 3 h after the administration of the probe using the IVIS Spectrum In Vivo Imaging System (PerkinElmer). Then, 3 h later, mice were sacrificed by CO_2_ exposure in a euthanasia chamber, and tumors and organs (lungs, liver, kidneys, spleen, and bladder) were immediately removed. Tumors and organs were analyzed immediately after harvesting in the IVIS system and finally processed for further analysis. **NB** was detected at 666 nm using an excitation wavelength of 636 nm. Fluorescence images were analyzed using the Living Imaging software from Caliper Life Sciences. The results are expressed as mean ± SD, and statistical analysis was performed by applying Two-way ANOVA with multiple comparisons using GraphPad software. A p-value below 0.05 was considered statistically significant and indicated with asterisks: *p < 0.05, **p < 0.0, ***p < 0.005, and ****p < 0.001.

### Evaluation of Senescence Burden in Tumors

X-Gal staining of tumors from 4T1 tumor-bearing mice treated or not with palbociclib was performed using the Senescence β-Galactosidase Staining Kit. Tumors were fixed in 4% paraformaldehyde (PFA) for 45–60 min at room temperature and then incubated overnight with the β-galactosidase staining kit at 37 °C (Cell Signaling, #9860S) following the manufacturer′s instructions. Tissues were post-fixed overnight in 4% PFA, embedded in paraffin, and cut into 5 μm slides. Besides, immunohistochemical staining of the proliferation biomarker Ki67 in tumor slides was performed. Tumors were fixed in PFA 4%, included in paraffin, and cut into 5 μm slides. Then, tumor slides were deparaffinized and rehydrated, and antigen retrieval was performed using 10 mM sodium citrate and 0.05% Tween 20 buffer at pH 6.0 for 30 min. Tumor sections were then incubated in blocking solution (5% horse serum, 0.3% Triton X-100 in 1 × PBS) for 1 h and incubated with Ki67 antibody (Abcam) at 4 °C overnight. Ki67 immunostaining was developed using 3,3-diaminobenzidine tetrahydrochloride (DAB), and nuclei were counterstained with hematoxylin. Sections were scanned in Leica Aperio Versa 200 equipment.

## Supporting Information Available

Synthesis and characterization (^1^H and ^13^C NMR and HR-MS) of the **NBGal** probe; HPLC-MS of the **NBGal** probe and **NB** fluorophore; UV and fluorescence of **NBGal** and **NB**; fluorescence of the **NBGal** probe and **NB** at different pH values and concentrations; experimental procedure for the limit of detection calculation; quantum yield measurements; cell viability assays with **NBGal**; validation of the probe in several senescent and non-senescent cell lines; and a table comparing recently published β-galactosidase probes (PDF)

## Abbreviations

CDK: cyclin-dependent kinases
FBS: fetal bovine serum
FDA: Food and Drugs Administration
HPLC-MS: high-performance liquid chromatography-mass spectrometry
IVIS: *in vivo* imaging system administration
NB: Nile blue
NIR: near infrared
NMR: nuclear magnetic resonance
PBS: phosphate-buffered saline
PFA: paraformaldehyde
ROS: reactive oxygen species
SA-βGal: senescence-associated β-galactosidase
SASP: senescence-associated secretory phenotype
TIS: therapy-induced senescence
