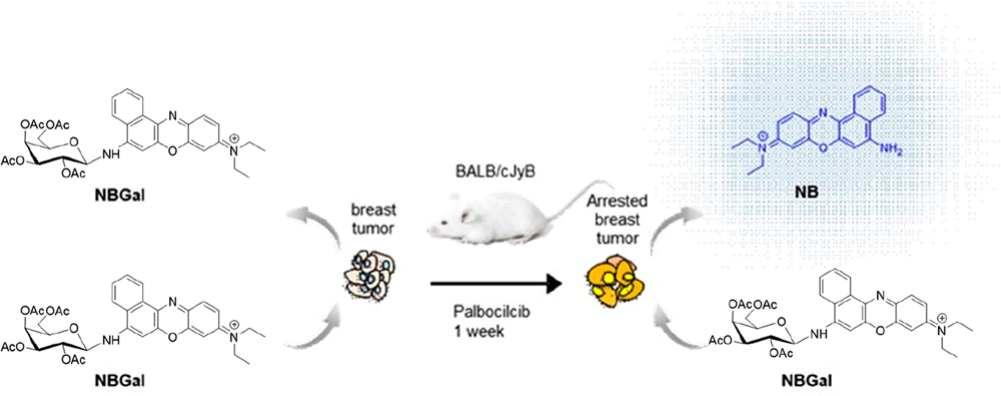# Correction to “β-Galactosidase-Activatable Nile Blue-Based NIR Senoprobe for the Real-Time Detection of Cellular Senescence”

**DOI:** 10.1021/acs.analchem.3c00131

**Published:** 2023-01-23

**Authors:** Beatriz Lozano-Torres, Alba García-Fernández, Marcia Domínguez, Félix Sancenón, Juan F. Blandez, Ramón Martínez-Máñez

We would like to correct the structure of the **NBGal** probe, which appeared in our original paper, because the configuration of the asymmetric carbons in the galactose unit are wrong. For this reason, we correct this error in the TOC and Abstract graphic, Scheme 1, and Figure 1a. Also, the structure of 2,3,4,6-tetra-*O*-acetyl-α-d-galactopyranosyl bromide in Figure 1a in our original paper is wrong and this fact is also corrected. In addition, we correct Figure 3c.

**Scheme 1:**
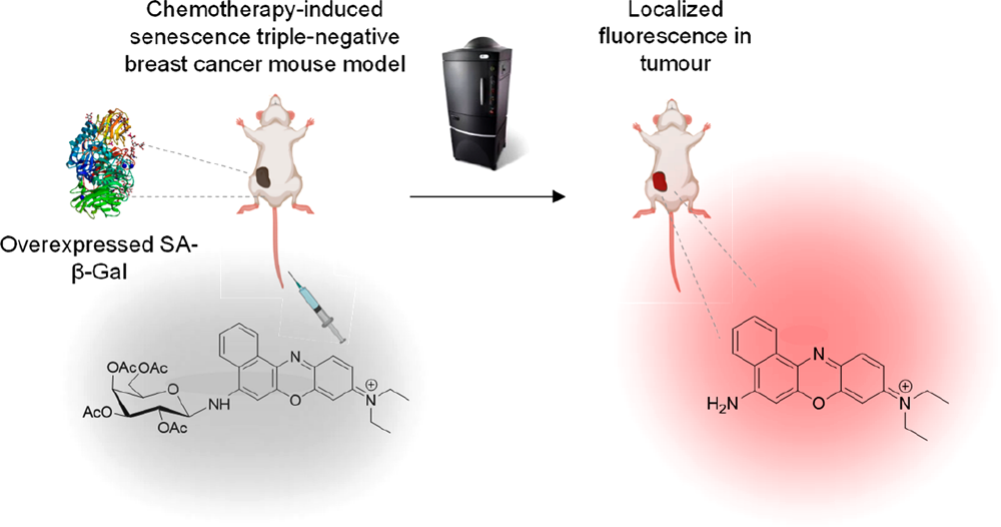
Representation of β-Gal-Activatable **NBGal** Probe for the *in Vivo* Monitoring of Cellular Senescence

**Figure 1:**
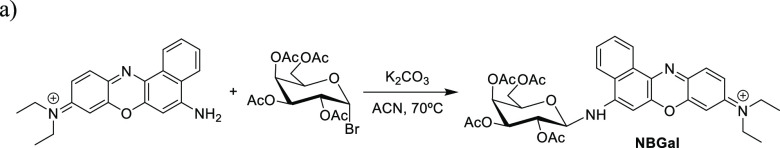
(a) Synthesis of **NBGal** probe.

**Figure 3:**
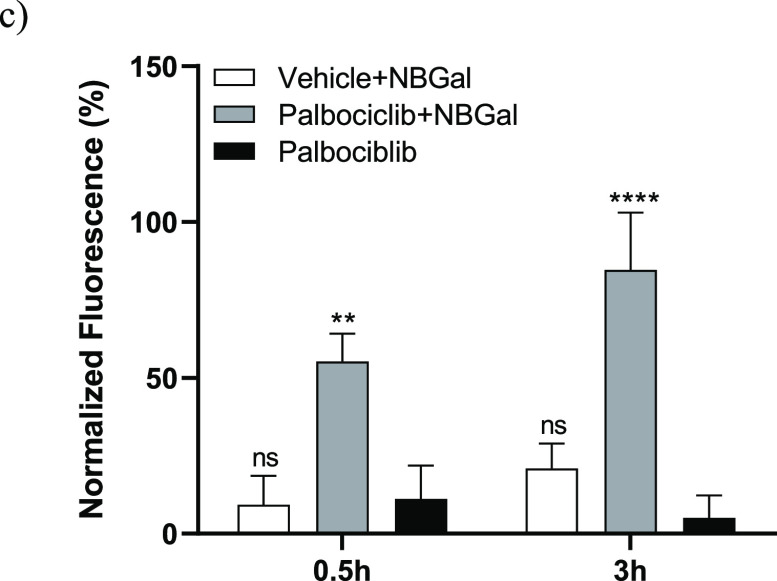
(c) Quantification of average radiance intensity from IVIS images in tumor zone shown in (b). The results are expressed as mean ± SD and statistical analysis was performed by applying two-way ANOVA with multiple comparisons (**p < 0.01 and **** *p* < 0.001).

Table of contents