# Supplementary material for: β-Galactosidase-Activatable Nile Blue-Based NIR Senoprobe for the Real-Time Detection of Cellular Senescence
Source: Anal Chem. 2022 Dec 29;95(2):1643–51. doi: 10.1021/acs.analchem.2c04766 (PMC9850349; doi:10.1021/acs.analchem.2c04766)
Supplement: Supplementary file 1 — ac2c04766_si_001.pdf [file ac2c04766_si_001.pdf]

Supporting Information

β-galactosidase-activatable Nile Blue-based NIR senoprobe for the real-time detection of cellular senescence

Beatriz Lozano-Torres,[§†¥‡] Alba García-Fernández,[§†¥?‡] Marcia Domínguez,[§¥] Félix Sancenón,[§† ¥?*] Juan F Blandez,[§ ¥?*] and Ramón Martínez-Máñez[§¥†?*]

§ Instituto Interuniversitario de Investigación de Reconocimiento Molecular y Desarrollo Tecnológico (IDM), Universitat Politècnica de València-Universitat de València, Camí de Vera S/N, Valencia, 46022, Spain.
† Unidad Mixta UPV-CIPF de Investigación en Mecanismos de Enfermedades y Nanomedicina, Universitat Politècnica de València, Centro de Investigación Príncipe Felipe, C/ Eduardo Primo Yúfera 3, Spain Valencia, 46012, Spain.
¥ CIBER de Bioingeniería, Biomateriales y Nanomedicina, Av. Monforte de Lemos, 3-5, Pabellón 11, Planta 0, Madrid, 28029, Spain.
? Unidad Mixta de Investigación en Nanomedicina y Sensores. Universitat Politècnica de València, IIS La Fe, Av. Fernando Abril Martorell, 10, Torre A 7ª planta, Valencia, 46026, Spain.

*Correspondence: rmaez@qim.upv.es, juablbaba@upvnet.upv.es, fsanceno@upvnet.upv.es
‡ Both authors contributed equally to this work

Table of contents

1.- Materials…………………………………………………………………….…...…S2

2.- Synthesis of **NBGal**……………………………………………………………...S2

3.- UV-visible and fluorescence spectra of **NBGal** and **NB**…………………………………S4

4.- Emission of **NBGal** and **NB** as a function of pH……………………………………………S5

5.- Fluorescence of **NBGal** and **NB** at different concentrations………………………………..S5

6.- Limit of detection (LOD) measurement…………………………………………….……S6

7.- NB and **NBGal** quantum yield measurements……………………………………………S6

8.- Cell viability assays……………………………………………………………………….S7

9.- Validation of **NBGal** probe in chemotherapeutic-induced senescence cancer models……...S8

Spectroscopic and sensing features of recently published β-galactosidase fluorescent probes…S9

1.- Materials.

Chemical reagents were obtained from Sigma–Aldrich. Phosphate-buffered saline (PBS, 0.01 M) and anhydrous solvents were purchased from Scharlab S.L. and used without further purification. Palbociclib was obtained from Selleckchem, and Dulbecco's modified Eagle medium (DMEM) and fetal bovine serum (FBS) were purchased from Sigma-Aldrich. Flat-bottom clear 96-well plates and CellTiter-Glo(R) Luminescent Cell viability kit were obtained from Promega. Senescence β-Galactosidase Staining Kit was obtained from Cell Signaling. Recombinant Human β-Galactosidase-1/GLB1 Protein (Human β-Gal) was purchased from R&D systems. The SK-Mel-103 (human melanoma) cancer cell line and 4 T1 (murine triple-negative breast cancer cells) were acquired from the American Type Culture Collection (ATCC). BALB/cByJ female mice were purchased from Charles River Laboratories, France.

Equipment. ^{1}H and ^{13}C NMR spectra were collected on a Bruker FT-NMR Avance 400 (Ettlingen, Germany) spectrometer at 300 K, using TMS as an internal standard. HPLC-MS were obtained with an Agilent 1620 Infinity II HPLC coupled to a mass spectrometer Agilent Ultivo equipped with a triple QTOF detector. Absorbance spectra were recorded in a JASCO V-650 while for fluorescence spectra was employed a JASCO FP-8500 fluorescence spectrophotometer. Luminescence was collected in a VICTOR multilabel plate reader (PerkinElmer). Confocal fluorescence images were taken on a Leica TCS SP8 AOBS. Images were analyzed using ImageJ software. Monitoring of fluorescence in animals was carried out in a IVIS Spectrum In Vivo Imaging System (PerkinElemer) and images were analyzed by using the Living Image software.

2.- Synthesis of NBGal

NB (417 mg/mmol, 208 mg, 0.5 mmol), acetobromo-α-D-galactose (411 mg/mmol, 616 mg, 1.5 mmol) and K_2CO_3 (138 mg/mmol, 414 mg, 3 mmol) were charged into a round bottomed flask. After purged with argon atmosphere, anhydrous acetonitrile (20 mL) was added, and the reaction was stirred for 4 h at 70 ºC. Afterward, solvent was removed under vacuum pressure to dryness. The residue was purified by flash chromatography on silica gel with ethyl acetate/hexane 1:10 v/v (1% NH_3) as eluent. **NBGal** was isolated as a purple-red solid (188 mg, 0.29 mmol, 58 % yield). ^{1}H-NMR (400 MHz, $CDCl_3$) δ 8.60 (d, J = 6.8 Hz, 1H), 8.24 (dd, J = 7.7, 1.4 Hz, 1H), 7.66 (td, J = 7.6, 1.6 Hz, 1H), 7.61 – 7.54 (m, 2H), 6.90 (s, 1H), 6.63 (dd, J = 9.0, 2.8 Hz, 1H), 6.44 (d, J = 2.6 Hz, 1H), 6.34 (s, 1H), 5.63 (d, J = 8.3 Hz, 1H), 5.36 (dd, J = 3.4, 1.2 Hz, 1H), 5.34 – 5.19 (m, 1H), 5.13 – 4.82 (m, 1H),3.42 (q, J = 7.2 Hz, 4H), 2.77 (t, J = 8.1 Hz, 1H), 2.53 (dd, J = 9.3, 6.8 Hz, 1H), 2.10 (s, 3H), 2.05 (s, 3H), 2.00 – 1.82 (m, 12H). ^{13}C-NMR (101 MHz, $CDCl_3$) ^{13}C NMR (101 MHz, $CDCl_3$) δ 170.68, 170.52, 170.40, 170.21, 156.41, 156.06, 150.09, 148.08, 146.86, 131.32, 130.49, 130.05, 125.83, 124.82, 123.73, 117.31, 108.80, 96.58, 90.77, 71.17, 70.51, 68.45, 67.40, 66.35, 45.07, 26.26, 25.57, 20.94, 20.80, 20.78, 12.75. HRMS: Calculated for $C_{34}H_{37}N_3O_{10}$ (M^+) 648.205 m/z; measured 648.255 m/z.

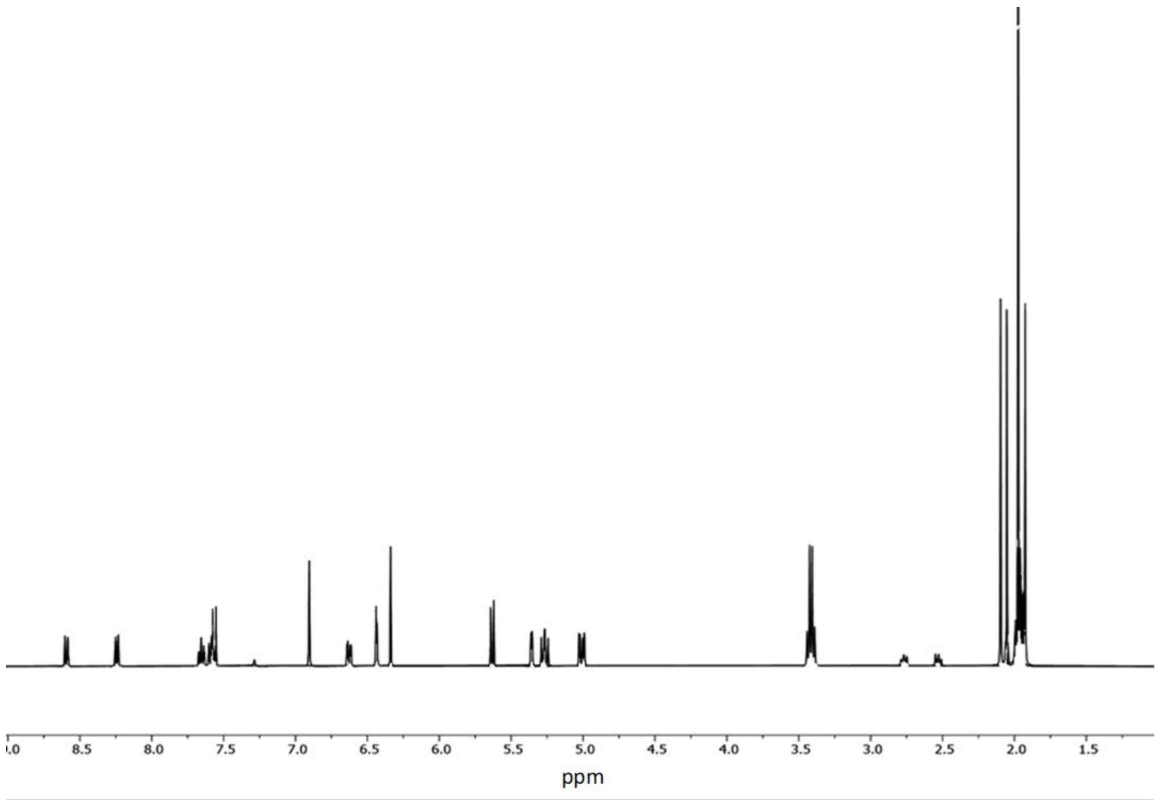

Figure S1. ^{1}H-NMR of **NBGal** probe in $CDCl_3$.

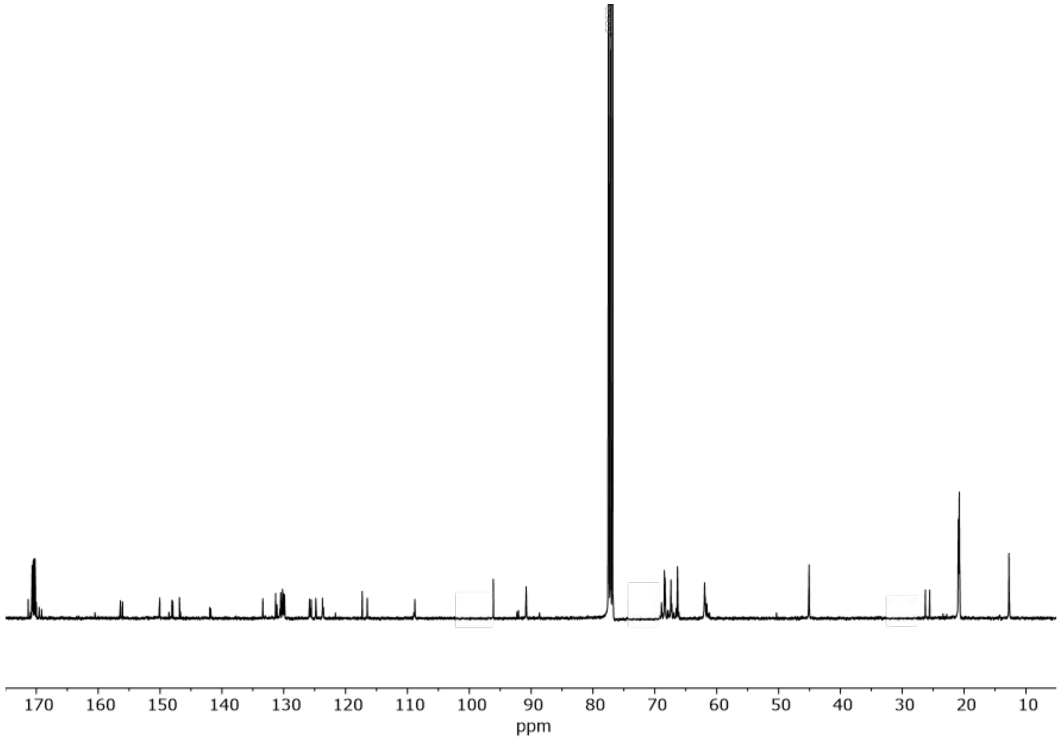

Figure S2. ^{13}C-NMR of **NBGal** probe in $CDCl_3$.

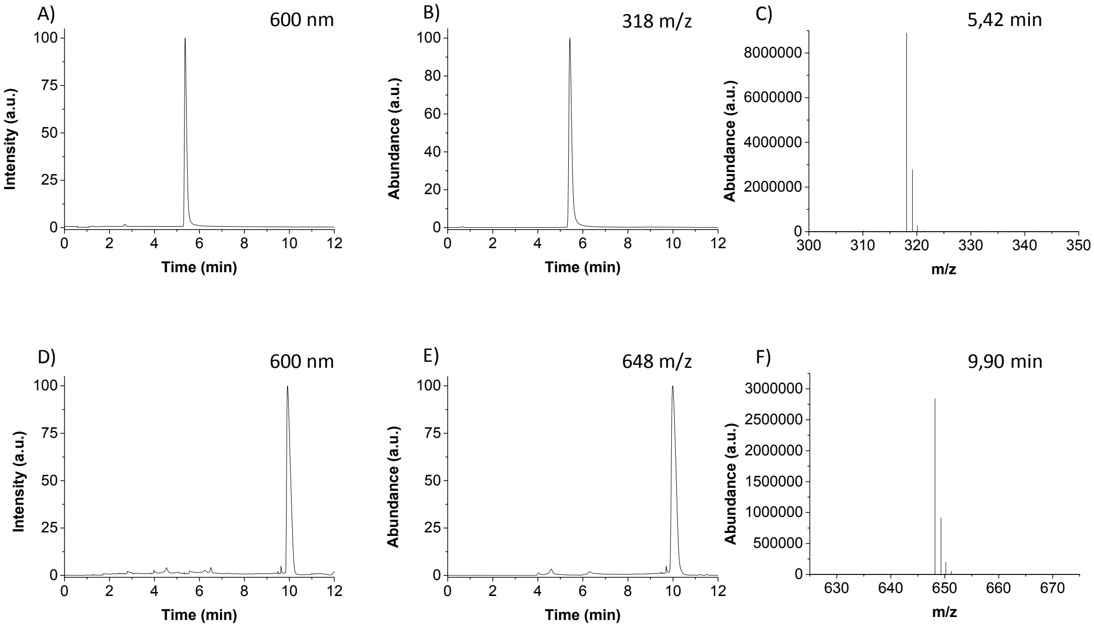

Figure S3. HPLC-MS of **NB** fluorophore and **NBGal** probe. (A) UV chromatogram of **NB** at 600 nm. (B). Single Ion Monitoring for **NB** molecular ion at 318 m/z. (C) Mass spectrum of **NB** obtained from the peak at 5.42 min showing 318 m/z value of $M+H^+$. (D) UV chromatogram of **NBGal** at 600 nm. (E). Single Ion Monitoring for **NBGal** molecular ion at 648 m/z. (F) Mass spectrum of **NBGal** obtained from the peak at 9.90 min showing 648 m/z value of $M+H^+$. using a gradient eluent method from H_2O-acetonitrile (30:70 v/v) to H_2O-acetonitrile (90:10 v/v) at 15 min with a flow rate 0.7 mL/min.

3.- UV-visible and fluorescence spectra of NBGal and NB

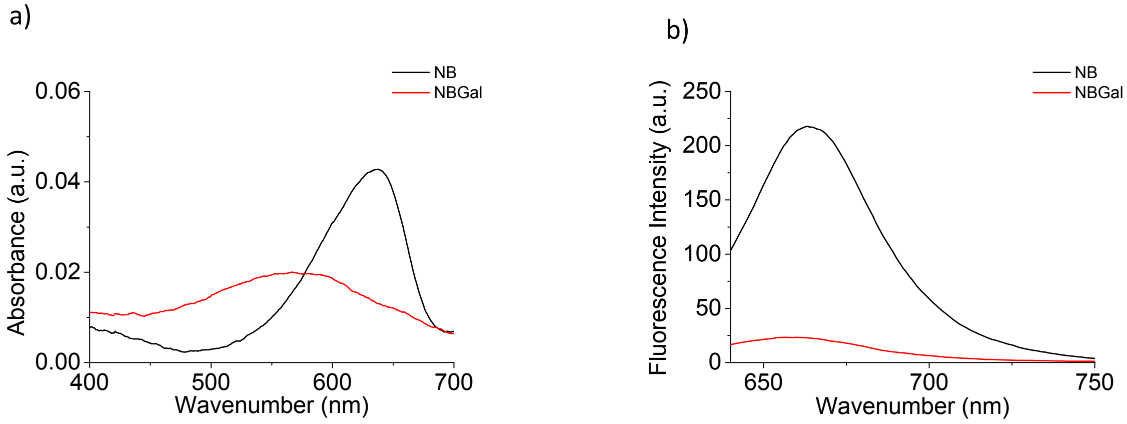

Figure S4. (a) UV-visble spectra of **NBGal** probe and **NB** fluorophore (1.0 x 10^{-6} M) in H_2O-DMSO (0.01%) pH7. (b) Fluorescence spectra of **NBGal** probe and **NB** fluorophore (1.0 x 10^{-6} M) in H_2O-DMSO (0.01%) pH 7 both upon excitation at 636 nm.

4.- Emission of NBGal and NB as a function of pH

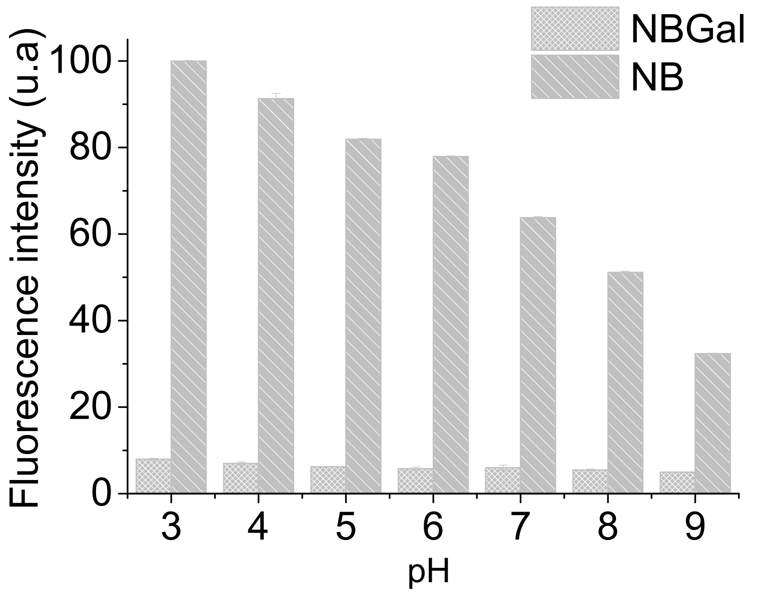

Figure S5. Normalized emission intensity at 666 nm (λ_{ex} = 636 nm) of **NBGal** and **NB** (1.0 x 10^{-6} M) in H_2O-DMSO (0.01%) solutions at pH 3, 4, 5, 6, 7, 8 and 9. Error bars represent SD (n=3).

5.- Fluorescence of NBGal and NB at different concentrations

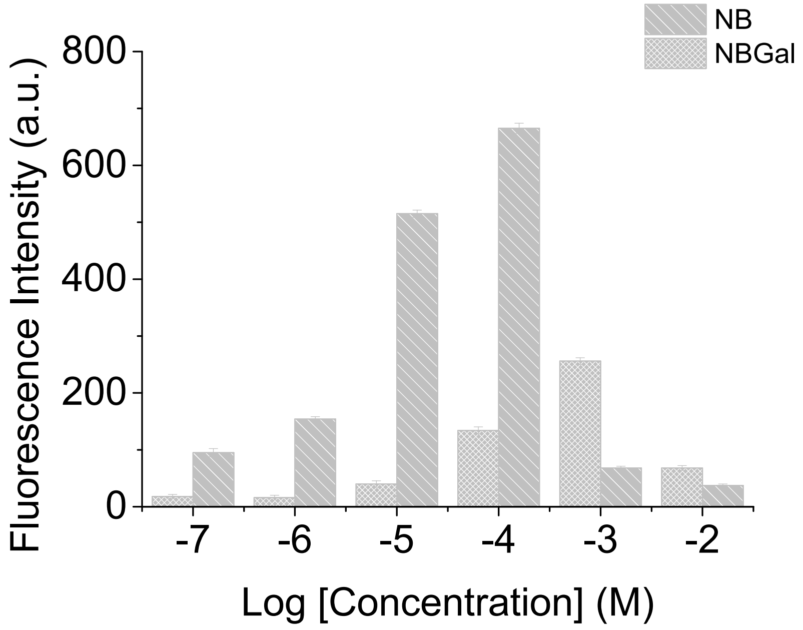

Figure S6. Emission intensity at 666 nm (λ_{ex} = 636 nm) of **NBGal** and **NB** in H_2O-DMSO (0.01%) solutions at pH 7 at different concentrations. Error bars represent SD (n=3).

6.- Limit of detection (LOD) measurement

Limit of detection for **NBGal** probe was obtained from the plot of fluorescence emission recorded at 666 nm (upon excitation at 635 nm) 60 min after addition of β-galactosidase enzyme (Figure S7). LOD was calculated using equation S1, where K=3; Sb is the standard deviation of the blank and m is the slope of the calibration curve. The resulting LOD for β-galactosidase enzyme was 6.86 ng/mL (2.33 U/mL).

$$LOD = K \times \frac{S_b}{m} \quad (S1)$$

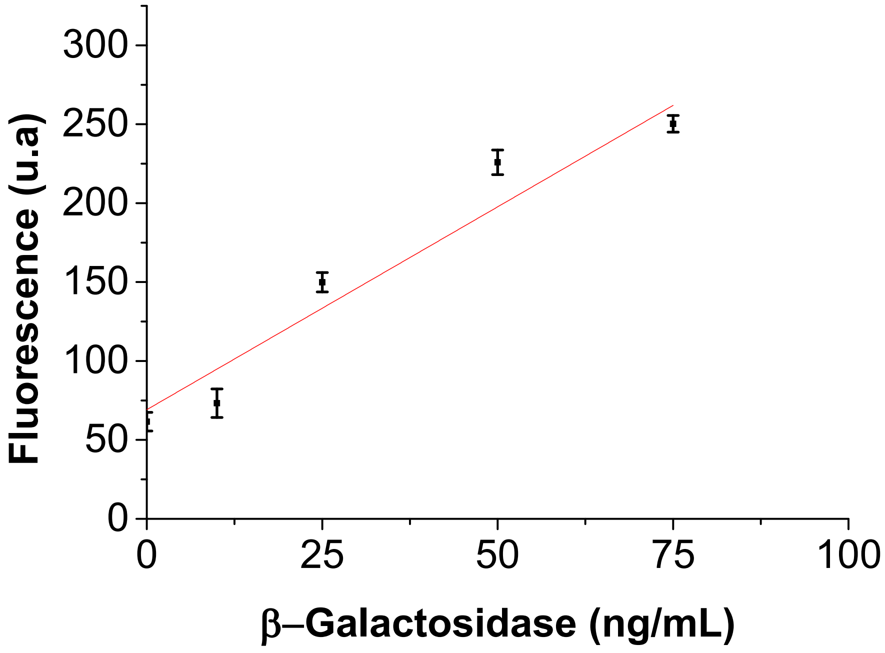

Figure S7. Calibration curve of **NBGal** probe in the presence of increasing amounts of β-galactosidase enzyme.

7.- NB and NBGal quantum yield measurements

The quantum yields of **NB** and **NBGal** in PBS (pH 7)-DMSO (0.01%) were measured using **NB** in water as standard (Φ = 0.01) applying the equation S2:

$$\frac{\Phi_x}{\Phi_s} = \frac{S_x}{S_s} \times \frac{1 - 10^{-A_s}}{1 - 10^{-A_x}} \times \frac{n_x^2}{n_s^2} \quad (S2)$$

where x and s indicate the unknown and standard solution, respectively, Φ is the quantum yield, S is the area under the emission curve, A is the absorbance at the excitation wavelength and n is the index of refraction.

8.- Cell viability assays.

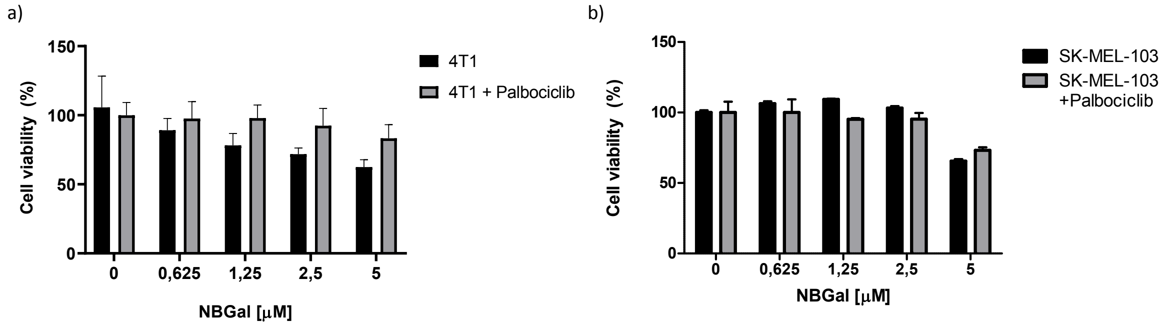

Figure S8. Cell viability assays in proliferative and senescent 4T1 (a) and SK-Mel-103 (b) cells, respectively in the presence of **NBGal** probe at different concentration for 48 h. The results are expressed as mean ± SD from three independent studies (n=3).

9.- Validation of NBGal probe in chemotherapeutic-induced senescence cancer models.

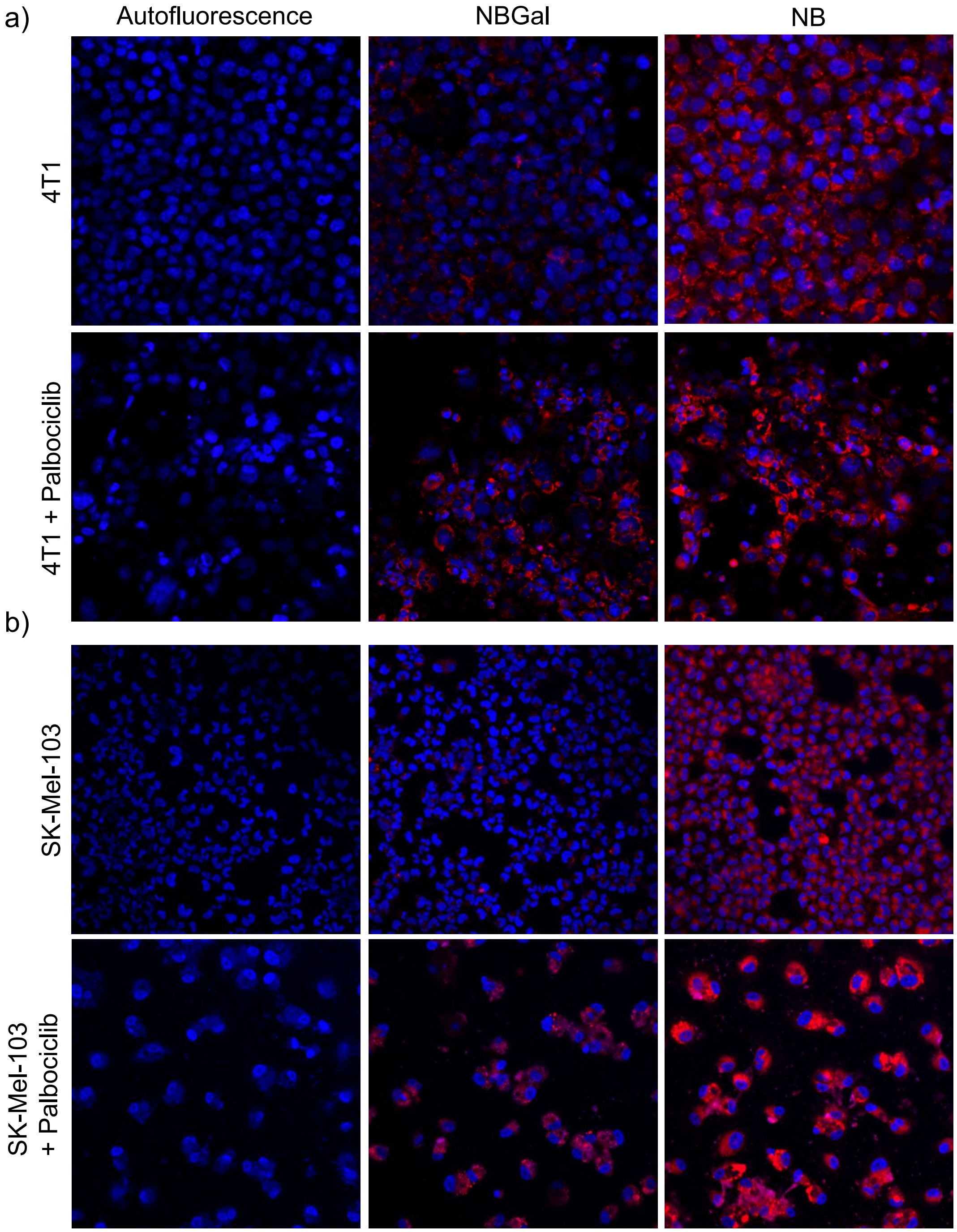

Figure S9. Confocal images of proliferating and senescent cells, after the treatment with Palbociclib, of triple-negative breast cancer 4T1 cells (S9a, up) and human melanoma SK-Mel-103 cells (S9b, down), respectively. From left to right: untreated cells as control, cells treated with the **NBGal** probe at 1.25 μM and **NB** dye in equivalent dose. Representative images obtained at 20x magnification using a Leica TCS SP8 AOBS confocal microscope.

Table S1. Spectroscopic and sensing features of recently published β-galactosidase fluorescent probes.

Probe	λ_{exc} (nm)	λ_{em} (nm)	*In vitro* studies		*In vivo* studies				Reference
			Cell line	Senescence induction	Animal model	Senescence induction	Two photon excitation	IVIS	
**AHGa**	750	540	SK-Mel-103	Palbociclib	SK-Mel-103 Xenograft	Palbociclib	Yes	No	*J. Am. Chem. Soc*. **2017**, *139*, 8808
**NIR-BG**	680	708	MCF7	Camptothecin or radiation	CT26 xenograft	Camptothecin	No	Yes	*Sci. Rep*. **2019**, *9*, 2021
			HeLa		HeLa xenograft				
**NIR-BG2**	650	709	HeLa	Camptothecin or radiation	HeLa xenograft	Camptothecin	No	Yes	*bioRxiv* **2020**, https://doi.org/10.1101/2020.03.27.010827
**HeckGal**	950	550	Sk-Mel-103	Palbociclib	4T1 orthotopic breast tumor	Palbociclib	Yes	Yes	*Anal. Chem*. **2021**, *93*, 3052
			4T1						
			A549	Cisplatin	Renal fibrosis	Folic acid			
			SK-Mel-103	Doxorubicin					
			BJ fibroblast						
**TR-G**	550	638	OVCAR-3	-	OVCAR-3 xenograft	Endogenous overexpression	Yes	Yes	*J. Mater. Chem. B* **2019**, *7*, 3431
**Gal-MB**	655	675	MDA-MB231	Palbociclib	-	-	No	No	*RSC Adv*. **2022**, *12*, 4543
**PGal-FA**	451	526	A549	Bleomycin	-	-	No	No	*ACS Sens*. **2022**, *7*, 1958
**NB-Gal**	636	666	SK-Mel-103	Palbociclib	4T1	Palbociclib	No	Yes	This work
			4T1						
